# Protocol for a James Lind Alliance priority setting partnership to identify the most important research priorities addressing respiratory health disparities affecting the Black community in the UK

**DOI:** 10.1136/bmjopen-2025-109741

**Published:** 2026-02-02

**Authors:** Kasope Wolffs, Melanie Etti, Ruby Zelzer, Priyanka Punja, Suzannah Kinsella, Erika Kennington, Adewale Adebajo

**Affiliations:** 1 School of Biosciences, Cardiff University, Cardiff, UK; 2 Department for Continuing Education, University of Oxford, Oxford, UK; 3 Reimagine Redefine, Nottingham, UK; 4 Asthma and Lung UK, London, UK; 5 James Lind Alliance, National Institute for Health Research, University of Southampton, Southampton, UK; 6 School of Health and Related Research, The University of Sheffield, Sheffield, UK; 7 School of Health and Related Research, National Institute for Health and Care Research Sheffield Biomedical Research Centre, Sheffield, UK

**Keywords:** Pulmonary Disease, Asthma, Pulmonary Disease, Chronic Obstructive, Health Equity, Health policy, Community Participation

## Abstract

**Abstract:**

**Introduction:**

Respiratory diseases affect millions of people in the UK, with a disproportionately high burden seen among many marginalised communities. They are the third leading cause of death in the UK and a major driver of morbidity, disability and healthcare service use. Many respiratory conditions cause debilitating symptoms and deterioration in patients’ health and quality of life over time, resulting in substantial increases in National Health Service (NHS) expenditure. Social inequalities, including occupational, housing and environmental disparities, have led to a disproportionate burden of respiratory disease among the Black community. For many Black people living in the UK, respiratory conditions have been under-recognised, misdiagnosed or inadequately treated, further contributing to disparities in health outcomes. Despite the need to address these urgent challenges, research in this area is fragmented and rarely informed by the views and opinions of those most affected. Research prioritisation provides a structured methodology to address this unmet need. The Equal Breath Priority Setting Partnership (PSP) aims to identify the 10 most urgent research priorities in respiratory health for people of Black heritage through meaningful collaboration with people with lived experience of respiratory disease, their caregivers and family members and the healthcare professionals caring for them.

**Methods and analysis:**

The top 10 research priorities for the Equal Breath PSP will be established using the James Lind Alliance (JLA) method. A steering group comprising approximately 12 people from key stakeholder groups will first be assembled to guide the PSP. Once the context and scope of the PSP has been agreed, the first survey will be developed and disseminated among stakeholder communities to identify evidence uncertainties. Data analysis of the survey responses will create summary questions and critical appraisal of available evidence will verify which of these are evidence gaps. A longlist of approximately 50 summary questions derived from the first survey will be shared with stakeholders in a second shortlisting survey. The highest ranking questions from this survey will be taken into a workshop where the top 10 research priorities will be established through a consensus process.

**Ethics and dissemination:**

This PSP employs the JLA methodology, which does not constitute research as defined by the Health Research Authority. Survey respondent data will be stored in accordance with UK General Data Protection Regulation by Asthma+Lung UK. The final 10 research priorities will be shared with funders, policymakers, professional bodies and relevant communities to inform future investment and promote equity in respiratory health.

**STRENGTHS AND LIMITATIONS OF THIS STUDY**

- This project employs the well-established James Lind Alliance methodology which has been utilised in more than 200 priority setting partnerships (PSPs) globally.
- The steering group comprises an interdisciplinary group of healthcare professionals, third sector representatives and members of the Black community in the UK with lived experience of respiratory conditions.
- Publication of the protocol outlining the project methods used enables transparency and reproducibility of the priority setting process.
- Language and low digital literacy or access may act as barriers to reaching members of our target population during the survey and consultation phases.
- To increase the accessibility of the PSP, the project will include in-person community engagement activities and use translators where appropriate.

## Introduction

 The burden of respiratory disease in the UK is significant. Respiratory conditions affect approximately one in five people across the country and are the third most common cause of death annually, surpassed only by cancer and cardiovascular disease.^1^ While significant gains have been made in cancer and cardiovascular disease outcomes over the last decade, outcomes from respiratory illnesses have failed to improve to the same extent, largely due to chronic research underfunding and a failure to address social inequalities that drive the development and progression of many of these conditions.^1 2^

The Black community has disproportionately poor respiratory health across a number of conditions compared with other racial groups in the UK. A study by Simms-Williams and colleagues, which analysed hospital admissions data between January 2017 and December 2019, found that Black people across all age categories (children, adolescents and adults) were at greater risk of asthma-related hospital admissions in the UK than any other race.^3^ Another study by Fry *et al* identified that Black Caribbean women in England had greater odds of being diagnosed with late stage non-small cell lung cancer than their white counterparts.^4^ Gayle and colleagues also found that the odds of a missed diagnosis of chronic obstructive pulmonary disease among Black patients were almost double that of White patients,^5^ further highlighting the challenges that members of the Black community in the UK face in accessing timely healthcare.

Social inequities, including disparities in housing and exposure to harmful respiratory irritants, also disproportionately affect the Black community in the UK.^6^ An analysis commissioned by the Greater London Authority found that people of Black and mixed heritage were most likely to live in areas of the city with illegal levels of air pollution.^7^ Additionally, figures from the Ministry of Housing, Communities and Local Government showed that 22% of Mixed White and Black Caribbean households and 14% of Black African households in England have problems with damp compared with only 4% of White households,^8^ further illuminating how many of these factors are interlinked. The deaths of 2-year-old Awaab Ishak, who died of a respiratory condition linked to chronic mould exposure in his home,^9^ and 9 year-old Ella Adoo-Kissi-Debrah, who was the first person in the UK to have ‘air pollution’ listed on her death certificate as a contributing factor towards her death,^10^ serve as sobering and poignant reminders of the consequences of these inequalities.

In 2021, NHS England launched the Core20PLUS5 initiative which seeks to reduce health inequalities among the most deprived 20% of the national population and marginalised groups with poor healthcare access and health outcomes, including members of Black and ethnic minority communities.^11 12^ Chronic respiratory conditions were highlighted among the initiative’s top five clinical areas of focus for both adults and children, underscoring its importance for the health and well-being throughout the life course.

Addressing the root causes of poor respiratory health of the Black community in the UK requires a deeper understanding of the complex interplay between the biological, social, environmental, systemic and structural factors that influence these outcomes. As such, targeted research which aims to identify and interrogate these interrelated factors is urgently needed. The Equal Breath Priority Setting Partnership (PSP), a collaborative project between the Medical Research Council Black in Biomedical Research Advisory Group, Asthma+Lung UK and the James Lind Alliance (JLA),^13^,^15^ seeks to determine the 10 most important questions for research to help address these inequities. Through an inclusive, iterative process, the PSP will co-develop a research agenda that centres voices and experiences from the Black community and the healthcare professionals who care for them, with the aim of guiding future research investments, informing evidence-based policy, and shaping future research efforts aimed at improving the respiratory health of Black people living in the UK.

## Methods and analysis

**Figure 1:**
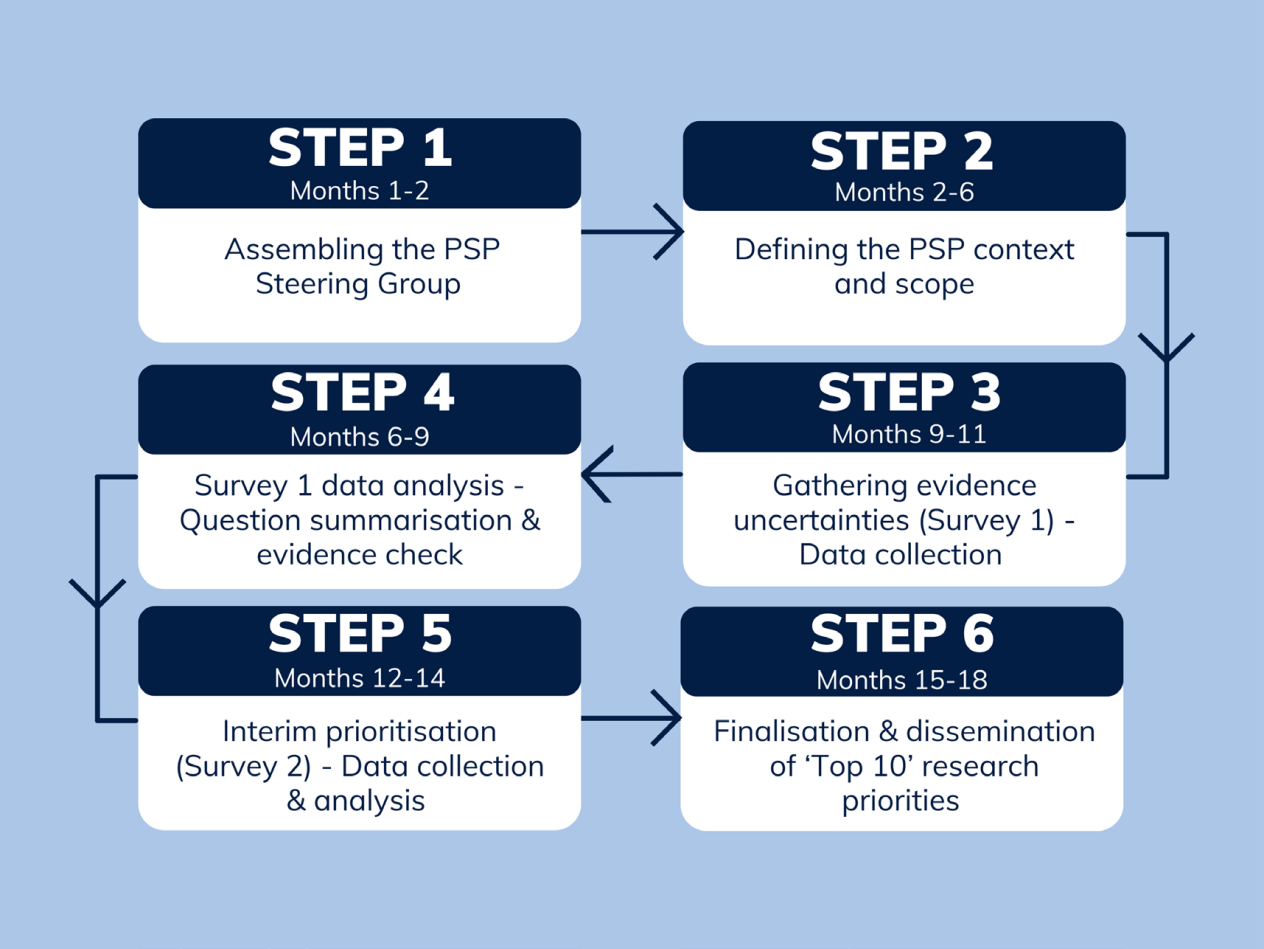
Flowchart showing the priority setting process for the Equal Breath PSP (in accordance with JLA methodology). JLA, James Lind Alliance; PSP, Priority Setting Partnership.

The Equal Breath PSP will be conducted in accordance with JLA methodology over a period of 12–18 months.^16^ The planned start and end dates are 31 March 2025 and 30 September 2026, respectively. The primary output will be the final list of agreed on top 10 research priorities addressing respiratory health disparities affecting the Black community in the UK. A flowchart summarising the priority setting process is shown in figure 1.

### Step 1: Assembling the PSP steering group

The PSP will begin with the establishment of a steering group (SG),^17^ over an 8-week period. The primary role of the SG is to drive the progress of the PSP by defining its context and scope, developing strategies through which to disseminate surveys, reviewing and interpreting the survey data and disseminating the project outputs.

SG members will be purposively selected to reflect a broad range of stakeholders and perspectives. This includes representation from regions across the UK, community organisations, a breadth of clinical specialties and healthcare roles and Black individuals with lived experience of a variety of respiratory conditions, including parents and caregivers. While not prescriptive, the suggested number of SG members is 12 people,^17^ although larger groups may be appropriate to ensure key stakeholder groups are represented. SG membership will be established through a structured recruitment process, during which professional, charity, community and grassroots networks will be engaged. To avoid a potential conflict of interest, representatives of the pharmaceutical industry will be excluded from joining this SG.^18^ A representation gap analysis will be carried out to assess the diversity of the group. Where gaps are identified, whether in relation to geography, lived experience, clinical background or community affiliation, further efforts will be made to recruit individuals who fulfil these shortcomings. Monthly virtual meetings, chaired by the JLA Adviser,^19^ will be held to ensure continued engagement and maintain project momentum.

### Step 2: Defining the PSP context and scope

Once established, the SG will first define the context and set the scope of the PSP supported by the JLA Adviser. The role of the JLA Adviser is to provide independent and neutral facilitation during SG meetings to ensure parity of opinion among SG members. Suggested areas relevant to the PSP will be proposed by SG members and consensus will be sought to ensure agreement among the majority of group members. Where there is disagreement regarding the appropriateness of an area for inclusion within the scope of the PSP, SG members will be invited to discuss further in order to achieve consensus.

The principal realms within which this PSP aims to generate research priorities are (i) to explore the root causes and contributing factors of respiratory health disparities affecting people of Black heritage in the UK, including biological, environmental, behavioural, occupation and social determinants of respiratory health, (ii) to understand and improve the experiences of diagnosis, treatment and clinical care for people of Black heritage with chronic respiratory conditions, chronic or recurrent lung infections and unexplained or undiagnosed respiratory symptoms, including exploring barriers to access to timely healthcare and assessing interventions designed to address these disparities and (iii) to interrogate the role of systemic racism, culture, communication and research inclusion in shaping respiratory health among the Black population in the UK. The PSP will consider research priorities relevant to scientific disciplines and the humanities, including questions pertaining to both paediatric and adult populations, and the impact of maternal health on the respiratory health of the developing fetus. The scope will also include the impact of COVID-19 within the Black community in the UK, including the impact of long COVID that is predominantly characterised by respiratory symptoms, but will not address non-respiratory sequelae of this infection. The full protocol for this PSP, including details of the context and scope of this PSP, will be made available on the JLA website.^15^

### Step 3: Gathering evidence uncertainties

After defining the context and scope of the PSP, the SG will oversee an initial consultation over a period of 12 weeks to gather questions and suggested areas of uncertainty in respiratory health of people of Black heritage in the UK. This process will involve the development and dissemination of a survey (Survey 1) by SG members. The survey will be made available in standard and easy read versions; the easy read version will be designed to enable participation from respondents at a range of literacy levels, including children. Survey 1 will include open-ended questions, allowing respondents to submit uncertainties and challenges around their respiratory health and potential solutions or interventions that might address these challenges. The survey will be piloted by the SG and a small group of stakeholders prior to wider dissemination to ensure accessibility. The proposed sample size for Survey 1 (100 adults with lived experience (including caregivers), 30 healthcare professionals and 20 children) is informed by consultations with community researchers and research organisations experienced in engaging Black communities and is considered sufficient to ensure diversity of perspectives for an exploratory prioritisation exercise.

Survey 1 will be developed using Typeform Software (Barcelona, Spain) and hosted on the Asthma+Lung UK website. Dissemination of the electronic survey will be through a combination of methods, including via social media, newsletters, respiratory health charities, community and faith groups, schools, barber shops, general practitioners (GP) surgeries and professional networks represented within the SG (such as Primary Care Respiratory Society and British Thoracic Society). Local dissemination within communities will be supported by Ageing Lifestyle in Blacks and Asians (ALIBSA), Community Gateway Cardiff, Multicultural Marketing Consultancy and Ndukauba CIC (Jollof Nights™). These activities will focus on major cities across the nation and regions with high populations of Black people (including London, Birmingham, Manchester, Leeds, Cardiff and Glasgow). National dissemination organisations include the Caribbean and African Health Network, Centre for Research Equity and School and Public Health Nurses Association. A short video will also be developed to explain the purpose of the project and guide participants through the survey.

Personal identifiable data will not be collected as part of Survey 1. Survey respondents will also be invited to provide demographic data (including age, gender, ethnicity and country of residence) on an optional basis. Respondents who express interest in Survey 2 or the final workshop will be directed via a unique link to a separate form where they may voluntarily provide an email address. These data will be stored separately from survey responses in accordance with the Asthma+Lung UK privacy policy. Anonymised demographic data from survey respondents will also be reviewed regularly by the SG to ensure broad stakeholder representation. Where needed, targeted outreach will be undertaken to improve participation from underrepresented groups.

Special consideration will be given to capturing the views of seldom-heard groups. This includes children and young adults aged under 30 years, Black men (who are typically underrepresented in health research^20^), people living in areas of high social deprivation and individuals with respiratory conditions that are rare within the British population such as idiopathic pulmonary fibrosis and pulmonary sarcoidosis. We will also seek to engage asylum seekers, refugees, individuals for whom English is not a first language or where cultural or communication differences may influence access to or trust in healthcare, as well as people experiencing homelessness and those who are digitally excluded. To maximise participation, Survey 1 will be offered in multiple formats. Respondents will have the option to complete the survey online or in person. Community organisations will host facilitated sessions to support individuals completing the survey in person. Translators will be made available where required to facilitate the inclusion of participants who do not speak English.

In order to explore key themes in greater depth and contextualise responses, we will also undertake in-person community engagement activities, including individual and roundtable discussions. Participants will be identified through our local community partners and purposive sampling to ensure diversity of perspective. 10 community-based engagement sessions will be organised across the UK at places of worship and community centres that predominantly serve Black communities. Each session will aim to engage approximately 10 individuals. While the primary output of these sessions will be completed survey responses, facilitators will document salient points arising from the discussions using brief anonymised quotes and annotated observations to support interpretation of the survey data. Sessions will not be audio- or video-recorded. Data from these sessions will be analysed thematically and any additional questions that emerge from these activities will be included to the list of evidence uncertainties.

### Step 4: Survey data analysis

Following closure of Survey 1, suggested evidence uncertainties will be analysed, categorised and refined into summary questions by the PSP information specialist, over a 12-week period. Analysis of Survey 1 data will be a combination of thematic analysis and a computational approach. For computational work, this will begin with data exploration to prepare summary statistics and understand the structure of the demographics and response frequencies of the dataset. Computational data processing will be undertaken in Python, using packages such as sentence_transformers, SciKitLearn, Hierarchical Density-Based Spatial Clustering of Applications with Noise (HDBSCAN), Uniform Manifold Approximation and Projection (UMAP) and Natural Language Toolkit (NLTK). Open text responses will be cleaned—that is, white spaces removed, all text converted to be not capitalised—and analysed, for example, to relate response length to demographics. Natural Language Processing (NLP) tools such as ‘all-MiniLM-L6-v2’, ‘all-mpnet-base-v2’ or another suitable package will be used to prepare data for clustering; this includes a minimum word limit for responses to include only meaningful statements and separation of long responses to convert each sentence into a single entry. Different clustering models (K-means, UMAP-HDBSCAN) and different hyperparameters will be evaluated to identify the most suitable clustering approach. The best-performing model will be used to generate clusters of questions. The output from this stage will be assessed by the PSP information specialist who will separately conduct thematic analysis on survey data. The questions from the computational approach and thematic analysis will be compared. Any iterative adjustments will be documented.

Following this process, members of the SG will review all summary questions to ensure they are distinct, addressable by research and understandable to all. Similar or duplicate questions will be combined where appropriate. Where questions are out of scope, they will be omitted from the process and compiled separately. Summary questions will then be compared against high-quality evidence (such as systematic reviews) by the PSP Information Specialist to determine whether they have been answered. The quality of the available evidence will be assessed using verified critical appraisal tools, such as those produced by Grading of Recommendations Assessment, Development and Evaluation (GRADE) and the Joanna Briggs Institute (JBI).^21 22^ The PSP Information Specialist will complete reporting documents which outline the process used to assess the certainty of each summary question and details of the types and sources of evidence reviewed. Full details of this strategy will be published on the JLA website for transparency and to allow researchers and other interested parties to understand how the PSP determined that the questions remain unanswered, as well as document any limitations in the methods used. Summary questions identified as knowledge gaps in the literature, or those which have not been adequately addressed by existing research, will be collated and included in the interim prioritisation survey (Survey 2).

The JLA Adviser will act as an observer to the process to ensure accountability and transparency. All submitted survey responses, including those which are out of scope, will be published on the JLA website following completion of the priority setting exercise to enable complete transparency of the full priority setting process.

### Step 5: Interim prioritisation

The interim prioritisation stage will further refine the longlist of verified unanswered summary questions generated during the initial consultation process into a shortlist suitable for prioritisation at the final workshop.

Following the conclusion of the Survey 1 data analysis, the SG will develop an interim prioritisation survey (Survey 2), inviting respondents to select the 10 most important summary questions from the longlist according to their personal views. Survey 2 will aim to recruit a similar sample size to Survey 1 to ensure sufficient representation across key stakeholder groups. Participation in the survey will be open to all individuals among the aforementioned groups, regardless of whether they took part in Survey 1. Responses to this survey will be sought over a period of 8 weeks. Once Survey 2 is closed, the SG will review the highest-ranked summary questions among people with lived experience, caregivers and healthcare professionals separately to prevent skewing due to unequal participant numbers across stakeholder groups. The results will be collated and the 25 highest-ranked research questions which represent the different stakeholder priorities will be taken forward to the final prioritisation stage. If the results do not produce a clear ranking order, the SG will decide on an equitable tie-breaker method (eg, a vote), considering the balance of themes and topics.

### Step 6: Finalisation of research priorities

The PSP will culminate in a final priority setting workshop, where consensus on the 10 foremost priorities for addressing respiratory health disparities affecting people of Black heritage in the UK will be reached among the key stakeholders engaged throughout the process. The workshop will be held in person with approximately 30 participants. A subgroup of the SG will oversee the recruitment of workshop participants to ensure balanced representation of stakeholder perspectives. The workshop will be facilitated by three JLA advisers, and the top 10 priorities will be agreed through a structured consensus decision-making process. The final priorities will initially be framed as broad thematic areas, then later developed into structured research questions using the Population, Intervention, Comparison, Outcome (PICO) format by the SG members. Once the final workshop has been concluded, the SG will move towards finalising the PSP process by engaging with and disseminating the results to all key stakeholders and partner organisations.

## Patient and public involvement

The involvement of people with lived experience is an integral part of the JLA PSP methodology as it ensures that the identified research priorities represent the views and opinions of affected communities. Central to this process is the broad recruitment and balanced inclusion of people with lived experience of respiratory disease, their family members and caregivers, alongside healthcare professionals in the SG, in accordance with JLA principles.^23^ The views of patient and public members of the SG will be incorporated throughout each step of the protocol to ensure that public-facing materials, including surveys, advertisements and project outputs, are comprehensible and easily accessible, and that evidence uncertainties derived from survey data are important to members of the relevant communities, as well as the research community. The patient and public members of the SG will also be involved in the governance of the project, with equal weighting given to the views of each member of the SG. The involvement of patients and the public throughout all aspects of the project will hopefully foster greater trust within the Black community in the UK, ensuring that the final research priorities are closely aligned with their needs and values. Full details of patient and public involvement in this project will be reported after completion of the project in accordance with Guidance for Reporting Involvement of Patients and the Public (GRIPP2) standards.^24^

## Ethics and dissemination

This PSP employs the JLA methodology which does not constitute research as defined by the Health Research Authority (HRA), confirmed by the HRA decision tool outcome.^25^ The survey and priority setting workshop participants in this project are not research participants. Participant informed consent is not required. All survey and workshop prioritisation data will be anonymised.

Personal identifiable data will be stored by Asthma+Lung UK in accordance with the UK General Data Protection Regulation (2016) as detailed within their privacy policy.^26^

Following the conclusion of the PSP, the SG will develop outputs to enable the highlighted research priorities to be shared with funding and professional bodies, policymakers and relevant stakeholders. The results will be published in a peer-reviewed journal and presented at regional and national conferences which focus on respiratory and population health to promote the research priorities among the scientific community. The UK National Institute of Health and Research and Medical Research Council will also be informed of the final 10 research priorities identified by the PSP to enable research proposals addressing these priorities to be considered by appropriate funding streams. A lay summary will also be produced to disseminate the results of this project among members of the public. Further consultation will be undertaken among SG members to decide additional ways to disseminate the results of this work within relevant communities, with consideration given to the development of infographics, animations and videos which may be disseminated through our partner organisations and via social media.

## Conclusion

Research which aims to address health disparities must incorporate the needs of the specific population group in order to advance healthcare equity. The JLA approach provides a transparent and inclusive process to ensure meaningful outcomes are achieved. By developing this respiratory research agenda in partnership with people of Black heritage in the UK with lived experience of respiratory disease and the healthcare professionals who care for them, this project ensures that future research addresses the most pressing questions from a seldom-heard community. Given the persistent inequities across multiple social determinants of respiratory health, we hope this work will also serve as a model for future research prioritisation efforts in other underserved or marginalised groups.
