# Supplementary material for: Protocol for a James Lind Alliance priority setting partnership to identify the most important research priorities addressing respiratory health disparities affecting the Black community in the UK
Source: BMJ Open. 2026 Feb 2;16(2):e109741. doi: 10.1136/bmjopen-2025-109741 (PMC12878385; doi:10.1136/bmjopen-2025-109741)
Supplement: Supplementary file 1 [file bmjopen-2025-109741.reviewer_comments.pdf]

PEER REVIEW HISTORY

BMJ Open publishes all reviews undertaken for accepted manuscripts. Reviewers are asked to complete a checklist review form and are provided with free text boxes to elaborate on their assessment. These free text comments are reproduced below.

ARTICLE DETAILS

Title (Provisional)

Protocol for a James Lind Alliance priority setting partnership to identify the most important research priorities addressing respiratory health disparities affecting the Black community in the UK

Authors

Wolffs, Kasope; Etti, Melanie; Zelzer, Ruby; Punja, Priyanka; Kinsella, Suzannah; Kennington, Erika; Adebajo, Ade

---

VERSION 1 - REVIEW

**Reviewer**	**1**
**Name**	**Poberezhets, Vitalii**
**Affiliation**	**National Pirogov Memorial Medical University**
**Date**	**06-Oct-2025**
**COI**	**None**

In this manuscript, the authors presented a protocol for a priority setting partnership to establish the ten most important research questions addressing disparities in respiratory health among the Black community in the UK.

The manuscript is well prepared and provides readers with clinically usefull information; however, I recommend highlighting whether you considered including participants with language barriers in this research.

**Reviewer**	**2**
**Name**	**Dauletbaev, Nurlan**
**Affiliation**	**Philipps-Universitat Marburg**
**Date**	**22-Oct-2025**
**COI**	**None**

Wolffs et al. present a protocol for a study whose aim is to identify 10 most urgent research priorities related to respiratory health of persons with Black heritage. The study protocol

utilizes established James Lind Alliance methodology. This methodology was previously used to iteratively refine the list of priorities in previous studies. In the context of this study, this methodology should enable iterative narrowing down of survey results from the most comprehensive to the most succinct. In addition to traditional descriptive statistical analyses, study protocol proposes utilization of techniques of Natural Language Processing, unsupervised machine learning (in particular, clustering), as well as Large Language Models (LLM). These analyses aim to identify potential hidden patterns within the research topics suggested by the employed surveys. Study protocol further aims to gather the suggestions from diverse stakeholder groups. In general, this is a well-written and well-argued manuscript.

Deficiencies are few, but they are still worthy addressing for this study protocol to achieve sufficient detailization.

First, descriptions of proposed steps are largely narrative. It is partly understandable as the manuscript is submitted as a study protocol. Still, some quantitative metrics would have been helpful. For instance, the size of a steering group (Step 1) or timeframe of survey 1 would have been indicated to enable a better appreciation. Obviously, a steering group of 10 members has a different dynamic in comparison to a group of 500 members. Another example of similarly vague statement is the following sentence: "Dissemination of the survey will be through a combination of local and national networks". This sentence, too, would have benefited from concrete examples of which networks will be targeted.

One of the major concerns is the inconsistency between the stated aim to keep surveys anonymous ("Personal identifiable data will not be collected unless respondents choose to share their email address") and the statement that "Respondents will have the option to complete the survey via alternative methods, such as video conferencing or telephone". The latter statement is found in the next paragraph on page 10. Is it possible that parts of survey 1 will allow deanonymization? Or could it be that these parts aim to target specific individuals and should, therefore, abstain from anonymization? This inconsistency could create substantial problems down the road. For instance, two different consent forms will need to be prepared to properly reflect different approaches to survey anonymization. Given potential employment of different approaches, a concern should be voiced about the risk of inadvertent deanonymization of the parts of the surveys that are meant to remain anonymized. This aspect may require targeted consultations with the local digital data protection office.

Some statements, while being correct when applied to the general population, may not be sufficiently precise in the context of the study focus on persons with Black heritage. For instance, the concept of "rare diseases" is based on the prevalence of these diseases in the general population. However, certain conditions can be enriched in specific subpopulations. An suitable example is sickle cell disease. The prevalence of this disease is much higher among the persons of West African and Caribbean descent. In contrast, cystic fibrosis,

another rare disease, is much less prevalent among persons with Black heritage. The authors should reflect on the use and applicability of employed terminology, especially in the context of this study. As already mentioned above, sufficient detailization of revised study protocol would be helpful.

It is an interesting approach to apply unsupervised machine learning to identify hidden patterns in survey responses. Unfortunately, also this section of the manuscript is not sufficiently detailed. The authors presumably know which Python library will be used. Therefore, the statement that “Data processing will be performed in Python” is not sufficiently concrete. Python is not a software but a programming language. Specific libraries exist to enable specific quantitative analyses in biomedical research, including for unsupervised algorithms. This should be properly acknowledged.

Moreover, the manuscript appears to conflate techniques of Natural Language Processing (NLP: Topic Modeling and Latent Dirichlet Allocation), unsupervised learning (clustering: which algorithms?), and supervised learning (decision trees and k-nearest neighbors) into the same analytical workflow. Both NLP and clustering are useful in the context of this study, especially when used prior to or in parallel with utilization of LLM. In contrast, plausibility of application of supervised learning (that is, machine classifiers) is not clear. Moreover, proposed application of SHAP (SHapley Additive exPlanation values) is not justifiable, at least, based on present description. Quantitation of SHAP values is a popular method to increase explainability of machine classifiers like the trees-based Random Forest algorithm. However, this study does not seem to have an aim to classify survey responses. Based current description, there is no obvious added value for application of machine classifiers (that is, supervised learning). The latter typically build “black box” models based on datasets with known classes (i.e., "labels"), which is not the case in this study.

As “Generative artificial intelligence”, the authors most likely mean an LLM. This part of study protocol should also be described with higher precision. Uploading survey results to a commercial LLM like ChatGPT or Gemini may inadvertently impact the anonymity of the survey. Moreover, all currently available LLM make errors and are known to “hallucinate”. This could inadvertently lead to biases in the analyses. The best approach would be to apply a locally stored open-source LLM. This will address the data privacy concerns. As for errors and “hallucinations”, this study protocol should preview reasonable safeguards.

VERSION 1 - AUTHOR RESPONSE

Reviewer: 1
Dr. Vitalii Poberezhets, National Pirogov Memorial Medical University
Comments to the Author:
In this manuscript, the authors presented a protocol for a priority setting partnership to establish the ten most important research questions addressing disparities in respiratory health among the Black community in the UK.
The manuscript is well prepared and provides readers with clinically usefull information;

however, I recommend highlighting whether you considered including participants with language barriers in this research.

Rebuttal: We thank the Reviewer for this important observation. We agree that inclusion of participants with language barriers is essential to ensuring equitable participation and representation within the PSP. We have now clarified within the Methods section how we plan to support respondents with language needs.
Specifically, we have added details on the provision of translators during in-person community engagement sessions and clarified that Survey 1 can be completed with facilitated support without collecting identifiable information. We have also strengthened the description of accessibility measures across survey formats. These revisions appear in the updated manuscript.
Line 278 -286: "We will also seek to engage asylum seekers, refugees, individuals for whom English is not a first language or where cultural or communication differences may influence access to or trust in healthcare, as well as people experiencing homelessness and those who are digitally excluded. To maximise participation, Survey 1 will be offered in multiple formats. Respondents will have the option to complete the survey online or in-person. Community organisations will host facilitated sessions to support individuals completing the survey in person. Translators will be made available where required to facilitate the inclusion of participants who do not speak English."

Reviewer: 2
Dr. Nurlan Dauletbaev, Philipps-Universitat Marburg, McGill University Faculty of Medicine and Health Sciences
Comments to the Author:
Wolffs et al. present a protocol for a study whose aim is to identify 10 most urgent research priorities related to respiratory health of persons with Black heritage. The study protocol utilizes established James Lind Alliance methodology. This methodology was previously used to iteratively refine the list of priorities in previous studies. In the context of this study, this methodology should enable iterative narrowing down of survey results from the most comprehensive to the most succinct. In addition to traditional descriptive statistical analyses, study protocol proposes utilization of techniques of Natural Language Processing, unsupervised machine learning (in particular, clustering), as well as Large Language Models (LLM). These analyses aim to identify potential hidden patterns within the research topics suggested by the employed surveys. Study protocol further aims to gather the suggestions from diverse stakeholder groups. In general, this is a well-written and well-argued manuscript.

Deficiencies are few, but they are still worthy addressing for this study protocol to achieve sufficient detailization.

Comment 1: First, descriptions of proposed steps are largely narrative. It is partly understandable as the manuscript is submitted as a study protocol. Still, some quantitative metrics would have been helpful. For instance, the size of a steering group (Step 1) or timeframe of survey 1 would have been indicated to enable a better appreciation. Obviously, a steering group of 10 members has a different dynamic in comparison to a group of 500 members. Another example of similarly vague statement is the following sentence: "Dissemination of the survey will be through a combination of local and national networks". This sentence, too, would have benefited from concrete examples of which networks will be targeted.

Rebuttal: We thank the Reviewer for the positive appraisal of our manuscript and for highlighting areas where greater specificity would enhance the clarity and completeness of the protocol.

We have addressed each of the points raised by incorporating additional quantitative detail to the methods (Steps 1, 3, 4 and 5), clearer examples in the relevant sections, and updating Figure 1 to incorporate the timeline. We appreciate the reviewer's suggestion to add these details; we believe the manuscript is now strengthened as a result.

• Steering group size (Step 1):

We agree that the size of the steering group is an important detail for understanding the dynamics of the PSP. We have included additional information to the manuscript.

Line 184-186: "While not prescriptive, the suggested number of SG members is 12 people[17], although larger groups may be appropriate to ensure key stakeholder groups are represented."

• Timeframe for Survey 1 (Step 3):

We have now included the planned timeframe for administration of Survey 1 in the Methods section to provide greater transparency about the sequencing of project activities.

Line 230-232: "After defining the context and scope of the PSP, the SG will oversee an initial consultation over a period of 12 weeks to gather questions and suggested areas of uncertainty in respiratory health of people of Black heritage in the UK."

• Concrete examples of local and national networks for survey dissemination (Step 3):

We have expanded the dissemination section to specify the exact local grassroots organisations and national networks that will support survey distribution. These additions address the reviewer's request for clearer guidance on the channels through which the survey will be promoted.

Lines 253–260: "Local dissemination within communities will be supported by ALIBSA (Ageing Lifestyle in Blacks and Asians), Community Gateway Cardiff, Multicultural Marketing Consultancy, and Ndukauba CIC (Jollof NightsTM). These activities will focus on major cities across the nation and regions with high population of Black people (including London, Birmingham, Manchester, Leeds, Cardiff and Glasgow). National dissemination organisations include the Caribbean and African Health Network (CAHN), Centre for Research Equity, and School and Public Health Nurses Association (SAPHNA)."

• Sample size for Survey 1 (Step 3) and Survey 2 (Step 5):

We have included the estimated sample size for both surveys.

Lines 240–245: The proposed sample size for Survey 1 (100 adults with lived experience (including caregivers), 30 healthcare professionals, and 20 children) is informed by consultations with community researchers and research organisations experienced in engaging Black communities, and is considered sufficient to ensure diversity of perspectives for an exploratory prioritisation exercise.

Lines 356–359: Survey 2 will aim to recruit a similar sample size to Survey 1 to ensure sufficient representation across key stakeholder groups. Participation in the survey will be open to all individuals among the aforementioned groups, regardless of whether they took part in Survey 1.

Comment 2: One of the major concerns is the inconsistency between the stated aim to keep surveys anonymous ("Personal identifiable data will not be collected unless respondents choose to share their email address") and the statement that "Respondents will have the option to complete the survey via alternative methods, such as video conferencing or telephone". The latter statement is found in the next paragraph on page 10. Is it possible that parts of survey 1 will allow deanonymization? Or could it be that these parts aim to target specific individuals and should, therefore, abstain from anonymization? This inconsistency

could create substantial problems down the road. For instance, two different consent forms will need to be prepared to properly reflect different approaches to survey anonymization. Given potential employment of different approaches, a concern should be voiced about the risk of inadvertent deanonymization of the parts of the surveys that are meant to remain anonymized. This aspect may require targeted consultations with the local digital data protection office.
Rebuttal: We agree that the initial wording may have implied an inconsistency between anonymous survey completion and the availability of assisted methods. We have now clarified the procedures in the protocol to ensure complete alignment with our anonymisation strategy.
Specifically, we have clarified that:
1. Survey 1 remains fully anonymous across all modes of completion.
2. Facilitators will not record any personal identifiers during assisted completion.
3. Respondents who wish to provide contact details for participation in Survey 2 or the final workshop will do so via a separate, unlinked form, ensuring no risk of deanonymisation of the main survey dataset.
4. Demographic data remain optional.

We also note the Reviewer's point regarding data governance. We confirm that this approach has been reviewed and approved in alignment with Asthma + Lung UK's privacy and data protection policies, as they serve as the data controller for this project.
Revisions addressing this issue have been incorporated into the manuscript in the Methods (Step 3). Lines 263–269: "Personal identifiable data will not be collected as part of Survey 1. Survey respondents will also be invited to provide demographic data (including age, gender, ethnicity, and country of residence) on an optional basis. Respondents who express interest in Survey 2 or the final workshop will be directed via a unique link to a separate form where they may voluntarily provide an email address. These data will be stored separately from survey responses in accordance with the Asthma + Lung UK privacy policy."

Comment 3: Some statements, while being correct when applied to the general population, may not be sufficiently precise in the context of the study focus on persons with Black heritage. For instance, the concept of "rare diseases" is based on the prevalence of these diseases in the general population. However, certain conditions can be enriched in specific subpopulations. An suitable example is sickle cell disease. The prevalence of this disease is much higher among the persons of West African and Caribbean descent. In contrast, cystic fibrosis, another rare disease, is much less prevalent among persons with Black heritage. The authors should reflect on the use and applicability of employed terminology, especially in the context of this study. As already mentioned above, sufficient detailization of revised study protocol would be helpful.
Rebuttal: We thank the Reviewer for this observation regarding the importance of precise terminology when describing disease prevalence within specific population groups. We agree that conditions such as sickle cell disease and cystic fibrosis illustrate how prevalence can vary significantly across populations and appreciate the emphasis on careful use of epidemiological terms.
After reviewing our Introduction, we note that the manuscript does not refer to rare diseases or use terminology that conflates general-population prevalence with prevalence within Black communities. All statements regarding disease burden and health inequalities are drawn directly from published UK data, and all examples provided (asthma admissions, late-stage lung cancer diagnosis, COPD misdiagnosis and environmental exposures) specifically relate to Black communities where this is indicated by the cited evidence.

In light of the Reviewer's comment, we have reviewed the section to ensure our terminology remains precise and population-appropriate, and we confirm that the current wording is accurate and fully supported by the referenced literature.

Comment 4: It is an interesting approach to apply unsupervised machine learning to identify hidden patterns in survey responses. Unfortunately, also this section of the manuscript is not sufficiently detailed. The authors presumably know which Python library will be used. Therefore, the statement that "Data processing will be performed in Python" is not sufficiently concrete. Python is not a software but a programming language. Specific libraries exist to enable specific quantitative analyses in biomedical research, including for unsupervised algorithms. This should be properly acknowledged.
Rebuttal: We are grateful to the Reviewer for requesting this clarification, which has strengthened the methodological transparency of the protocol.
In response, we have expanded the Methods section to clearly outline the text-processing and clustering libraries that will be used for unsupervised machine learning analyses.
We now specify that:
• Processing of text responses before clustering will be conducted using text-embedding models such as 'all-MiniLM-L6-v2' or 'all-mpnet-base-v2' from the Python package 'sentence_transformers' or another suitable package.
• Clustering analyses will employ packages, including SciKitLearn, HDBSCAN, UMAP and NLTK.
• The manuscript now emphasises that Python functions as the programming environment, and that specific libraries will enable the relevant computational steps. These revisions have been added to the manuscript on Lines 306–323:
"Analysis of Survey 1 data will be a combination of thematic analysis and a computational approach. For computational work this will begin with data exploration to prepare summary statistics and understand the structure of the demographics and response frequencies of the dataset. Computational data processing will be undertaken in python, using packages such as sentence_transformers, SciKitLearn, HDBSCAN, UMAP and NLTK. Open text responses will be cleaned – i.e., white spaces removed, all text converted to be not capitalised – and analysed, e.g. to relate response length to demographics. Natural language processing (NLP) tools such as 'all-MiniLM-L6-v2', 'all-mpnet-base-v2' or another suitable package will be used to prepare data for clustering; this includes a minimum word limit for responses to include only meaningful statements and separation of long responses to convert each sentence into a single entry. Different clustering models (K-means, UMAP-HSBSCAN) and different hyperparameters will be evaluated to identify the most suitable clustering approach. The best performing model will be used to generate clusters of questions. The output from this stage will be assessed by the PSP Information Specialist who will separately conduct thematic analysis on survey data. The questions from the computational approach and thematic analysis will be compared. Any iterative adjustments will be documented."

Comment 5: Moreover, the manuscript appears to conflate techniques of Natural Language Processing (NLP: Topic Modeling and Latent Dirichlet Allocation), unsupervised learning (clustering: which algorithms?), and supervised learning (decision trees and k-nearest neighbors) into the same analytical workflow. Both NLP and clustering are useful in the context of this study, especially when used prior to or in parallel with utilization of LLM. In contrast, plausibility of application of supervised learning (that is, machine classifiers) is not clear. Moreover, proposed application of SHAP (SHapley Additive exPlanation values) is not justifiable, at least, based on present description. Quantitation of SHAP values is a popular method to increase explainability of machine classifiers like the trees-based Random Forest

algorithm. However, this study does not seem to have an aim to classify survey responses. Based current description, there is no obvious added value for application of machine classifiers (that is, supervised learning). The latter typically build “black box” models based on datasets with known classes (i.e., "labels"), which is not the case in this study.
Rebuttal: We confirm that the computational approach in this project is unsupervised. To provide clarity, the data processing pipeline will include four key parts:
1. Data exploration to prepare summary statistics and understand the structure of the full dataset (text responses and demographics).
2. Text data exploration to understand the data structure of the open text responses.
3. Text data processing (natural language processing, NLP), which includes data cleaning, identification, testing and evaluation of clustering procedures using the most suitable model to generate clusters uncertainties. This will be unsupervised. Clustering methods we will explore are K-Means clustering, HDBSCAN and UMAP-HDBSCAN.
4. Thematic analysis by a researcher coding and theming survey data with sentiment analysis will be used alongside human evaluation of the output from stage 3.
These steps have now been clearly reflected in the revised manuscript (Lines 309–317).

Comment 6: As “Generative artificial intelligence”, the authors most likely mean an LLM. This part of study protocol should also be described with higher precision. Uploading survey results to a commercial LLM like ChatGPT or Gemini may inadvertently impact the anonymity of the survey. Moreover, all currently available LLM make errors and are known to “hallucinate”. This could inadvertently lead to biases in the analyses. The best approach would be to apply a locally stored open-source LLM. This will address the data privacy concerns. As for errors and “hallucinations”, this study protocol should preview reasonable safeguards.
Rebuttal: We thank the Reviewer for this timely observation. We agree that the Reviewer’s comments on commercial models and hallucinations are valid.
In this revised protocol, GenAI is not used. If Gen AI had been used, only local SLMs that do not share data or requests with external agents would have been employed and safeguards for hallucinations would have included:
1. Clear documentation of processes;
2. Clear documentation of GenAI outputs;
3. Human input and comparison of original data with GenAI outputs;
4. Iterative adjustments to the GenAI process through a ‘human-in-the-loop’ approach;
5. Performing topic and emotional semantic analysis on both original data and GenAI outputs to compare assignments and validate retention of meaning.

VERSION 2 - REVIEW

**Reviewer**	**2**
**Name**	**Dauletbaev, Nurlan**
**Affiliation**	**Philipps-Universitat Marburg**
**Date**	**04-Jan-2026**
**COI**	

The authors have submitted a detailed and thoroughly reworked revision that addresses all previously raised comments
